# Advancing treatment choices: CDK4/6 inhibitor switching in HR+/HER2- metastatic breast cancer

**DOI:** 10.1016/j.breast.2025.103875

**Published:** 2025-01-10

**Authors:** Paola Zagami, Angela Esposito, Beatrice Taurelli Salimbeni, Pier Paolo Maria Berton Giachetti, Roberta Scafetta, Matteo Lambertini, Massimo Di Maio, Giuseppe Curigliano, Carmen Criscitiello, Saverio Cinieri

**Affiliations:** a Department of Oncology and Hematology, University of Milano, Milan, Italy; b Division of New Drugs and Early Drug Development, European Institute of Oncology IRCCS, Milan, Italy; c Department of Medical Oncology, Campus Bio-Medico University of Rome, Rome, Italy; d Department of Internal Medicine and Medical Specialties (DiMI), School of Medicine, University of Genova, Genoa, Italy; e Department of Medical Oncology, U.O. Clinica di Oncologia Medica, IRCCS Ospedale Policlinico San Martino, Genova, Italy; f Department of Oncology, University of Turin, Division of Medical Oncology, Ordine Mauriziano Hospital, Turin, Italy; g Medical Oncology Division and Breast Unit, Senatore Antonio Perrino Hospital, ASL Brindisi, Brindisi, Italy

**Keywords:** Breast cancer, CDK4-6i, Metastatic treatment and endocrine-based therapy

## Abstract

**Purpose:**

CDK4/6 inhibitors (CDK4/6i) use has revolutionized the treatment of hormone receptor-positive/human epidermal growth factor receptor 2 negative (HR+/HER2-) metastatic breast cancer. The choice of a specific CDK4/6i may be influenced by adverse events (AEs). Recently, the Italian Medicines Agency (AIFA) approved the possibility of switching between CDK4/6i for unacceptable toxicity. This study explores oncologists' experiences and future perspectives on CDK4/6 inhibitor switching following this new approval.

**Methods:**

With the support of the Italian Association of Medical Oncology (AIOM), we conducted a survey among 92 oncologists to assess the impact of AIFA's approval on patient management.

**Results:**

The survey showed that 48 % of participants were not surprised regarding AIFA's decision, with 76 % of respondents believing that this opportunity would significantly influence their treatment choices, enhancing AEs management for patients. Yet, 49 % of respondents emphasized the need for more real world evidence on CDK4/6i switch safety and efficacy. 96 % of respondents reported discontinuation rates between 0% and 25 % of patients, with constipation and hematological toxicity being the most frequent treatment discontinuation reasons. The oncologists prescribing CDK4/6i switch reported that most of these patients were in first line treatment (85 %) and the most common second CDK4/6i most frequently initiated was palbociclib (69 %), then abemaciclib (17 %) and ribociclib (14 %). Among those who started the second CDK4/6i at full dosage, 66 % of patients didn't require a dose reduction.

**Conclusion:**

Our survey highlights the importance of allowing CDK4/6i switching, thus likely prompting oncologists to adapt their treatment choices, leading to better AEs management for improving patients’ outcome.

## Highlights

• CDK4/6 inhibitors use have revolutionized hormone receptor-positive/HER2-negative metastatic breast cancer treatment.
• The possibility of switching between CDK4/6 inhibitors for unacceptable toxicity has been recently approved.
• Based on this approval, oncologists change their treatment choice enhancing adverse events management for patients.

## 1 Introduction

Breast cancer (BC) is the most common cancer among women worldwide [1]. Clinically, it can be classified based on the presence or absence of hormone receptors (HR) and/or human epidermal growth factor receptor 2 (HER2) into three main subtypes: hormone receptors positive (HR+)/HER2-; HER2-positive (HER2+), and triple negative BC (TNBC) if lacking both HER2 and HR expression. HR + BC is the most prevalent BC subtype, accounting for about 70–80 % of all BCs [2].

The growth and proliferation of HR + breast cancer cells are dependent on the binding of estrogens to their receptors. Endocrine therapy (ET), including drugs inhibiting the estrogen receptor (ER) pathway, has demonstrated efficacy in HR + BC. The development of different ET including selective estrogen receptor modulators (SERMs), aromatase inhibitors (AIs), and selective estrogen receptor degraders (SERDs) entailed an improvement in both survival and quality of life of patients with HR + metastatic BC (MBC) [3].

Over the past decades, with a better understanding of HR + BC biology, novel biological agents have been investigated such as inhibitors of the cyclin-dependent kinase 4/6 (CDK4/6i), namely palbociclib, ribociclib and abemaciclib. CDK4/6 plays a key role in cell proliferation through the G1/S transition inactivating the retinoblastoma (Rb) tumor suppressor protein. Thus, CDK4/6i selectively trigger cell cycle arrest in Rb-competent cells [4]. Palbociclib, ribociclib and abemaciclib, in combination with ET, changed the clinical practice of HR+/HER2- MBC treatment [5].

Many phase III trials investigating CDK4/6i plus ET demonstrated an improvement in progression free survival (PFS) with mixed results in overall survival (OS) in advanced or metastatic HR+/HER2- BC [[6], [7], [8], [9], [10], [11], [12], [13], [14], [15], [16], [17], [18], [19]]. Abemaciclib has also been approved as adjuvant treatment for high-risk HR+/HER2- BC and positive results with the use of adjuvant ribociclib have also been recently published [[20], [21], [22]].

The addition of a CDK4/6i to ET outperformed both AIs and fulvestrant alone in either first or subsequent lines of therapy. Most prospective trials documented positive results, regardless of several clinical factors (e.g. menopausal status, endocrine-sensitive status, visceral involvement, and others). The use of abemaciclib in combination with fulvestrant, in the MONARCH-2 study, showed a median PFS and OS of 16.4 and 46.9 months compared to 9.3 and 37.3 months of fulvestrant alone in the treatment of pre- and post-menopausal women with HR + MBC [8,11,15]. In the treatment of premenopausal or perimenopausal patients with HR + MBC, ribociclib with goserelin and either AIs or tamoxifen showed a median PFS of 23.8 months compared with 13.0 months in the placebo group and an OS at 42 months of 70.2 % in the ribociclib group versus 46 % in the placebo group [12,13]. In postmenopausal patients, ribociclib plus letrozole showed a median PFS of 25.3 months compared to the 16.0 months for placebo plus letrozole [9] and an OS of 63.9 months versus 51.4 months of the placebo group after a median follow-up of 6.6 years [14]. In the MONALEESA-3 study, ribociclib in combination of fulvestrant resulted in a PFS of 20.5 months versus 12.8 months in the placebo group [16]. In detail, patients that received ribociclib plus fulvestrant as first-line treatment had a median PFS of 33.6 months compared to 19.2 months in the placebo group, and showed an OS of 57.8 % compared to 45.9 % in the placebo group at 42 months [17]. The use of palbociclib and letrozole in postmenopausal patients instead showed a median PFS of 27.6 months compared to 14.5 months in the placebo group, but failed to reach a statistically significant OS (53.9 months for palbociclib plus letrozole vs 51.2 months of placebo plus letrozole), as well as abemaciclib in MONARCH-3 trial [18,19].

CDK4/6i–based treatments are able to significantly reduce the risk of death by 21 % in an endocrine-resistance setting and 27 % in endocrine-sensitive setting [23]. While currently there is no available data comparing these agents, an ongoing trial, HARMONIA (NCT05207709), is comparing the effect of endocrine therapy with either palbociclib or ribociclib in first-line patients with metastatic HR+/HER2- BC with HER2-enriched intrinsic molecular subtype [24]. Although the three CDK4/6i have reported similar PFS benefit in HR+/HER2- MBC, they show pharmacokinetic differences that are reflected in their dosing regimen and toxicities (Table 1). These differences may also explain (at least to some extent) the conflicting results observed with the three drugs in terms of OS in the metastatic setting as well as in the adjuvant setting.

**Table 1:** Differences in efficacy, side effects and dosing regimen across the three CDK4-6i.

CDK4/6 Inhibitor	Efficacy (e.g., PFS, OS)	Common Side Effects	Adverse effects from clinical studies led to the EMA CDK4-6i approval (any grade, G3)	Dosing Regimen
Palbociclib	- Improved PFS - not statistically significant difference in OS (with AI or fulvestrant)	Neutropenia, Fatigue	75–83 %,56–58 % 37–41 %,2–3%	125 mg daily, 3 weeks on/1 week off
Ribociclib	- Similar PFS to others - Statistically significant improvement in OS (with both fulvestrant and AI)	Neutropenia, QTc prolongation, Liver function alteration	65–75 %,43–51 % 2–10 %,2–5% 20–27 %,5–14 %	600 mg daily, 3 weeks on/1 week off
Abemaciclib	- Similar PFS - Slightly significant improvement in OS (with fulvestrant) - not statistically significant better OS (with AI)	Diarrhea, Neutropenia, abdominal pain	87-83 %,10–14 % 49-46,26–27 % 37-33,3-2%.	150 mg twice daily, continuous

### 1.1 Similarities and differences in pharmacokinetics

The three CDK4/6i show a similar pharmacokinetic profile, being rapidly absorbed and distributed, then mainly metabolized by CYP3A4 enzyme. However, treatment schedules are similar for palbociclib and ribociclib, with 3 weeks on/1 week off schedules (125 mg and 600 mg daily, respectively), while abemaciclib is given in a continuous schedule of 150 mg twice daily [4,25].

The difference in treatment schedules is mainly to reduce haematological toxicities of palbociclib and ribociclib, whereas preclinical studies that display drug absorption saturation exist for abemaciclib, supporting the twice-daily dosing regimen [26,27]. Moreover, the continuous abemaciclib exposure with sustained CDK4-6 inhibition induces the BC cells to a permanent cell cycle arrest leading to senescence. Contrarily, palbociclib and ribociclib induce BC cells quiescence with rebound induction of DNA synthesis [26,28].

Abemaciclib also shows a higher lipophilicity compared to the other CDK4/6is, that results in a theoretical ability to penetrate breast tissue and the blood-brain barrier [25]. Preclinical studies confirmed this hypothesis evaluating the presence of abemaciclib in both plasma and cerebrospinal fluid resulting from systemic treatment [29]. Moreover, research on human xenograft models showed an improved effect of abemaciclib in decreasing brain tumor growth, compared to palbociclib [30].

### 1.2 Adverse events and quality of life

Common adverse events (AEs) related to the three CDK4/6i are hematologic toxicities, especially neutropenia (mostly asymptomatic) and fatigue as reported in the pivotal clinical trials (Table 1) and in real world analyses with lightly lower incidence (fatigue 8 %, neutropenia 46 %) [31]. Cytopenia is an on-target effect of CDK4/6i that leads to a cytostatic effect on neutrophil precursors resulting in pharmacological quiescence that is rapidly reversible following withdrawal of the agent [32]. Specific drug-related toxicities are peculiar to each CDK4/6i.

For abemaciclib, gastrointestinal toxicity (diarrhea, nausea and abdominal pain), along with thromboembolic events, are peculiar and more common in the first months of therapy, while manageable with dose adjustments and concomitant medication [32]. Palbociclib, on the other hand, is typically characterized by hematologic toxicity [15,33,34]. Specific toxicity related to ribociclib is the prolongation of the QTc interval that, although occurring in a small proportion of patients in the phase III trials, requires ECG monitoring during the first 2 cycles [17,24,[35], [36], [37]]. Ribociclib may also result in hepatotoxicity which requires monitoring of liver function tests (LFTs) at baseline and during therapy [32]. Dose reductions and modifications are recommended for the management of hematological and non-haematological toxicities resulting from CDK4/6i treatment [38]. In the PALOMA 2 and 3 trials, approximately 5 % of patients needed a treatment dose reduction in cycle 1 or 2, achieving up to 40 % during the other cycles. Similar percentage of dose reduction during CDK4/6i treatment was described in MONALEESA-7, -2, and -3 trials as well as in MONARCH2 trial. The median time to dose reduction was 2–3 months from the start of CDK4-6i. In adjuvant monarchE trial 43 % of patient receiving abemaciclib needed dose reduction of whom the approximately 12 % during the first two months. The first approach to manage toxicity should consist in reducing the dose of the CDK4/6i, which does not compromise efficacy [[39], [40], [41], [42]].

For unacceptable toxicity, despite the dose reduction, the CDK4/6i treatment must be discontinued. In particular, a higher rate of permanent discontinuation is reported with abemaciclib (20 %) due to diarrhea, compared with palbociclib and ribociclib (7.5 % each), mainly due to myelosuppression [7,43,44]. The lower withdrawal rate with palbociclib and ribociclib might be linked to the intermittent dosing regimen, that during the treatment-free period allows the bone marrow cells to recover [45].

Recently, the Italian Medicines Agency (Agenzia Italiana del Farmaco, AIFA) approved the strategy of switching from one CDK4/6i to another in case of unacceptable toxicity, effectively expanding the treatment options for patients with MBC. This approval has opened new opportunities for patient care, ensuring a prolongment of an effective treatment, improving therapeutic management. In light of these developments and with the support of the Italian Association of Medical Oncology (AIOM), we report here the results of the survey conducted among Italian oncologists to understand the impact of the AIFA approval in enhancing the management of CDK4/6 inhibitor treatment and its toxicity.

## 2 Methods

### 2.1 Data source and study design

The survey collected information on CDK4/6i experience from 92 medical oncologists. Medical practitioners across Italy were recruited via email and reminders posted on the AIOM official website. No monetary incentives were provided.

Via 20 closed-ended questions, this study examined the experience of medical practitioners' with CDK4/6i toxicities and causes related to treatment discontinuation. It also investigates medical practitioners’ perspectives towards the recent decision of AIFA on the possibility to switch the CDK4/6i in case of observed toxicity. Participants provided their informed consent.

### 2.2 Data analysis

Data were analysed with descriptive statistics for average and median rates using Excel. Data from Table 1 were further analysed with weighted average to consider the occurring frequency of adverse events, weighting events from more frequent to less frequent (∗10, ∗7.5, ∗5, ∗2.5, ∗0.5). Frequencies were then normalized to percentages and plotted. Data visualization was performed with the open-source software LabPlot2 (https://labplot.kde.org/).

## 3 Results

A total of 92 Italian medical oncologists participated in the survey.

### 3.1 CDK4/6i treatment discontinuation due to toxicity

**Fig. 1:**
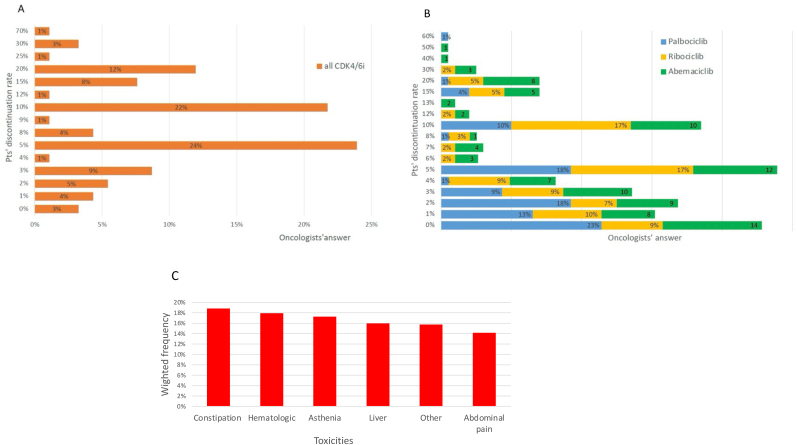
Survey answers on CDK4/6i discontinuation. Percentage of patients that needed discontinuation due to toxicity of all CDK4/6i (A), palbociclib (blue), ribociclib (yellow) or abemaciclib (green) (B), based on oncologists' experience. C) Types of unacceptable toxicities that most frequently led to discontinuation, weighted by their occurrence frequency.

The data from the questionnaire revealed that the range of CDK4/6i discontinuation rates varied from none to 70 %, with an average and median rate of 10 % and 8 %, respectively. Among the respondents, 96 % reported that 0%–25 % of their patients discontinued treatment due to constipation and hematological toxicity, the most frequent reasons cited. Further breakdown showed that 24 % of respondents reported a CDK4/6i discontinuation rate of 5 % of patients, 22 % reported 10 %, and 12 % reported 20 % of patients (Fig. 1A). Regarding the percentage of patients who had to interrupt palbociclib treatment due to toxicity, the questionnaire responses reported a discontinuation range from 0 % to 60 % (average rate of 4 % and median rate of 2 %). Among these responses, 23 % of respondents indicated 0 % of discontinuation cases, while 18 % reported discontinuation rates of 2 % and 5 % of patients (Fig. 1B, *blue*). A lower percentage of discontinuation due to toxicity was reported for ribociclib, ranging from 0 % to 30 % (average rate of 7 % and median rate of 5 %). In detail, 9 % indicated 0 % discontinuation and 17 % reported a rate of 5 % of patients (Fig. 1B, *yellow*). For abemaciclib, oncologists reported similar discontinuation rate to palbociclib, ranging from 0 % to 50 %, with an average rate of 8 % and a median rate of 5 %0.14 % of respondents reported 0 % discontinuation, and 12 % reported a rate of 5 % of patients (Fig. 1B, *green*). Table 2 reports the toxicity that led to CDK4/6i treatment discontinuation using a ranking of the frequency from 1 to 6.

**Table 2:** Toxicity frequency stratified from 1 (most frequent) to 6 (less frequent).

Toxicity	1	2	3	4	5	6
Constipation	39 %	8 %	2 %	4 %	17 %	29 %
Asthenia	3 %	33 %	24 %	15 %	16 %	9 %
Hematological toxicity	22 %	14 %	17 %	17 %	14 %	15 %
Hepatic toxicity	12 %	20 %	17 %	18 %	15 %	17 %
Abdominal pain	3 %	22 %	17 %	26 %	24 %	8 %
Other toxicities	21 %	4 %	22 %	18 %	13 %	22 %

Weighting the results by the occurrence frequency, constipation resulted to be the most frequent cause of inacceptable toxicity leading to discontinuation, followed by hematological toxicity. For 8 % of the oncologists, abdominal pain was the least frequent cause of treatment discontinuation (Fig. 1C).

### 3.2 Influence of CDK4/6i switch on initial drug choice

**Fig. 2:**
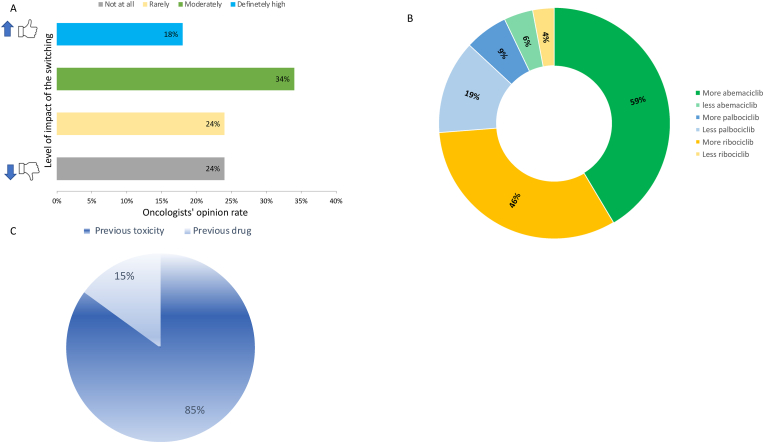
Oncologists' perspectives on the impact and possibility of CDK4/6i switching strategy on future treatment decisions. A) Level of influence of the possibility of CDK4-6i switching on the choice of the first CDK4/6i; B) Different oncologists' choice of first prescribed CDK4/6i after the switch strategy approval; C) factors that will influence the choice of the second CDK4/6i. Percentages from (B) and (C) were calculated based on a positive answer (blue, green and yellow) to question (A).

The survey showed that only for the 24 % of the 92 responders the possibility of changing CDK4/6i in case of unacceptable toxicity would not influence their initial drug treatment choice (Fig. 2A). Among the respondents who are likely to change their treatment choice, the preferred choice of CDK4/6i will reflect an increased abemaciclib use (59 % of oncologists) (Fig. 2B).

The primary factor that would influence the choice of the second CDK4/6i is the type of toxicity associated with the first CDK 4/6i. Among the 92 responders, 85 % indicated that they would base their second choice on the specific type of toxicity experienced with the initial CDK4/6i (Fig. 2C).

### 3.3 Reception of AIFA's decision and evidence to support CDK4/6i switch

**Fig. 3:**
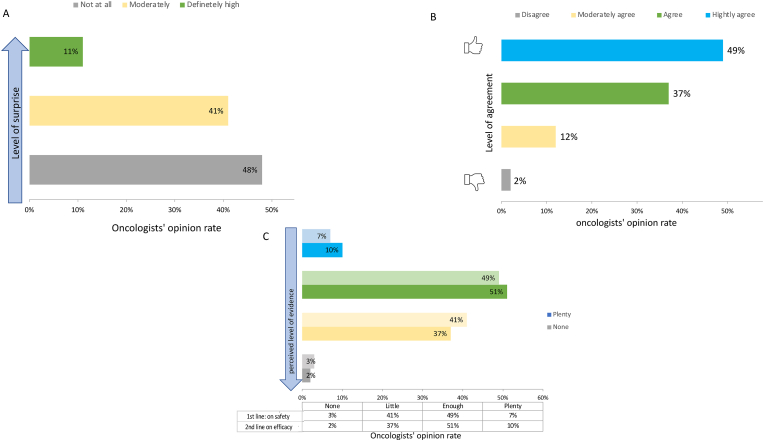
Answers regarding AIFA switch decision. A) Level of surprise regarding the decision; B) Level of agreement with the decision; C) Perceived level of evidence available on safety and efficacy regarding the CDK4/6i switch.

Only 11 % of responders indicated that was not expecting the decision of CDK4/6i switch made by AIFA, 41 % were quite surprised, whereas 48 % of participants were not surprised at all (Fig. 3A). AIFA choice met a high level of agreement in 49 % of responders, a good level of agreement in the 37 %, an adequate level of agreement in the 12 % and only a 2 % of disagreement (Fig. 3B). About the evidence supporting the *safety* of switching strategy of CDK4/6i, the percentage of oncologists who reported that there is plenty, enough, little and no evidence was of 10 % 51 %, 37 % and 2 % respectively (Fig. 3C). Similar results were reported about the evidence supporting the efficacy of changing CDK4/6i. 7 % of participants stated there is plenty of evidence, 49 % believed that there is enough evidence, little and no evidence were reported by the 41 %, and 3 % of oncologists, respectively (Fig. 3D).

### 3.4 Experience with CDK4/6i switch and future perspectives

**Fig. 4:**
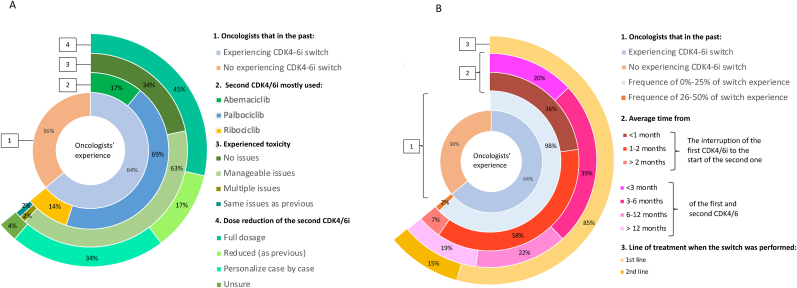
Experience regarding CDK4/6i switch. A) regarding the second CDK4-6i used. From circle 1 to 4. Oncologists who performed CDK4/6 switching (1); CDK4-6i mostly used after switching (2); experienced toxicity issues after the CDK4/6i switch (3) dose reduction of the second CDK4/6i if it was initiated at full dosage (4) B) regarding general experience in time and toxicity. From circle 1 to 3. Oncologists who performed CDK4/6 switching and in which percentage had experience (1); average time from the interruption of the first CDK4/6i to the start of the second one (grade of red) and of the first and second CDK4/6i (grade of pink) (2); Line of treatment when the switch was performed (grade of yellow) (3).

Regarding the starting dosage of the second CDK4/6i in a patient who was receiving the interrupted CDK4/6i at a reduced dosage, 45 % of participants would start with the full dosage, 17 % would start at the same reduced level as the previous drug, 34 % indicated that the decision would depend on the individual case, and 4 % were unsure and wanted more data before deciding (Fig. 4A). 64 % of the oncologists reported that they already used the strategy of CDK4/6i switching, with a percentage of switches ranging from 1 % to 30 % (average of 7 % and a median of 5 %). The majority (98 %) of the responses fell within the 0–25 % range, with 37 % of respondents reporting a 5 % switch and 20 % reporting a 10 % switch (Fig. 4B). Among the participants who made switches, the second CDK4/6i that was most frequently initiated was palbociclib (69 %), with abemaciclib (17 %) and ribociclib (14 %) being less used (Fig. 4A). Regarding the toxicity experience with the second CDK4/6i, 34 % of the participants did not experience any issue, 63 % experienced manageable issues, 2 % experienced multiple issues requiring discontinuation and 2 % experienced the same issues as with the first CDK4-6i (Fig. 4A). The most common time interval between discontinuing the first CDK4/6i due to toxicity and starting the second one was between one and two months (58 %), followed by less than a month (36 %), or after two months (7 %) (Fig. 4B). The average treatment duration within the first and the second CDK4/6i was less than 3 months (20 %), more than 3 months but less than 6 months (39 %), more than 6 months but less than 1 year (22 %) or more than a year (19 %) (Fig. 4B). At the time of the switch, 85 % of patients were in the first treatment line and 15 % of patients were in the second line or beyond (Fig. 4B). Among those who started the second CDK4/6i at the full dosage, 66 % of patients did not require a dose reduction and 34 % required a dose reduction.

## 4 Discussion and conclusions

CDK4/6 inhibitors represent the preferred first-line therapy for patients with HR+/HER2-metastatic breast cancer. The three different drugs demonstrated similar results in progression-free survival (PFS) but mixed results in overall survival (OS), with the choice of the preferred CDK4/6 inhibitor largely depending on toxicity profiles [46]. Palbociclib and ribociclib are noted for more hematologic toxicities, while abemaciclib presents more gastrointestinal toxicity. Key factors such as the risks of diarrhea and grade 3/4 neutropenia are major considerations in both oncologist and patient preferences [32]. Additionally, comorbidities, concomitant medications, costs, and access play crucial roles in the choice of CDK4/6 inhibitors. Dose reductions are common in pivotal trials and do not appear to compromise efficacy; however, toxicity can still occur, prompting a switch to another CDK4/6 inhibitor [47].

In a matching-adjusted indirect comparison of quality of life comparing ribociclib and abemaciclib with aromatase inhibitors, ribociclib showed advantages in managing appetite loss, diarrhea, fatigue, and arm symptoms [48]. AIFA's approval for switching between CDK4/6 inhibitors in case of unacceptable toxicity has enhanced the ability to prolong CDK4/6 inhibitor treatment, improving compliance and toxicity management. Despite these advances, limited data support continuing CDK4/6 inhibitor therapy beyond progression. Trials such as BioPER and PALMIRA have explored the efficacy and safety of continuing palbociclib with a different endocrine therapy agent beyond progression on a prior palbociclib-based regimen [48,49]. Recently an interim analysis of post-MONARCH trial showed improved PFS with abemaciclib plus fulvestrant as second line after progression on CDK4-6i plus AI. 59 %, 33 % and 8 % of these patients received palbociclib, ribociclib and abemaciclib as prior CDK4-6i [49]. However, it is important to consider that different outcomes can be expected with continuing (switching) CDK4-6i in case of unacceptable toxicity and in case of progression, because patient can have benefit from CDK4-6i if unacceptable toxicity occurs.

Our survey highlighted that constipation and hematologic toxicity were primary reasons for treatment discontinuation, whereas abdominal pain was less frequently a cause. Real-world studies support these findings, indicating that hematologic and hepatic toxicity are common reasons for discontinuation of the first CDK4/6 inhibitor, particularly ribociclib [50].

The introduction of the option to switch CDK4/6 inhibitors has been anticipated to significantly influence the management of HR+/HER2-metastatic breast cancer, with a notable impact on initial drug choice heavily influenced by the type of experienced toxicity. This emphasizes the importance of managing adverse effects to optimize treatment outcomes.

## 5 Future research directions

While current data on CDK4/6 inhibitors have significantly enhanced our understanding and management of HR+/HER2-metastatic breast cancer, gaps remain that warrant further investigation.

Future studies should focus on the long-term impacts of these treatments, monitoring patients for extended periods to gather data on survival, quality of life, and late-emerging side effects.

Considering the genetic and metabolic diversity across different populations, it is critical to assess the universal applicability of trial findings. Research involving diverse demographic groups could uncover population-specific efficacy and safety profiles, leading to more personalized treatment approaches. Moreover, after AIFA's approval CDK4-6i switching, the need to collect real-world data for the efficacy and safety of this approach in case of unacceptable toxicity, became urgent in order to better understand the main toxicities and the long duration of CDK4-6i treatment as reported in our survey.

Head-to-head trials comparing the three main CDK4/6 inhibitors in various settings could provide deeper insights into their optimal use and help clarify situations in which one drug might be preferred over others due to its efficacy, side effect profile, or ease of administration.

Investigating the mechanisms by which tumors develop resistance to CDK4/6 inhibitors will be crucial for developing next-generation therapies or combination strategies to prolong treatment effectiveness.

As the use of CDK4/6 inhibitors becomes more widespread, assessing their cost-effectiveness and financial impact on patients within different healthcare systems is vital.

These research areas are crucial not only for enhancing our understanding of CDK4/6 inhibitors but also for improving patient outcomes through more informed and tailored treatment strategies. Further research in these areas could lead to significant advancements in the management of metastatic breast cancer.

## CRediT authorship contribution statement

**Paola Zagami:** Writing – review & editing, Writing – original draft, Visualization, Validation, Methodology, Formal analysis, Data curation. **Angela Esposito:** Writing – review & editing, Visualization, Validation. **Beatrice Taurelli Salimbeni:** Writing – review & editing, Visualization, Validation. **Pier Paolo Maria Berton Giachetti:** Writing – review & editing, Visualization, Validation. **Roberta Scafetta:** Writing – review & editing, Validation, Supervision. **Matteo Lambertini:** Writing – review & editing, Visualization, Validation. **Massimo Di Maio:** Visualization, Validation. **Giuseppe Curigliano:** Writing – review & editing, Visualization, Validation, Supervision. **Carmen Criscitiello:** Writing – review & editing, Writing – original draft, Visualization, Validation, Supervision, Formal analysis, Conceptualization. **Saverio Cinieri:** Writing – review & editing, Visualization, Validation.

## Disclosures

All disclosers are outside the submitted work.

CC: consultancy/advisory role/speaker bureau: Pfizer, Novartis, Lilly, Roche, MSD, Seagen, Gilead, AstraZeneca, Daiichi Sankyo. Institutional research funding: Seagen, Gilead. These companies had no role in the design of the study; in the collection, analyses, or interpretation of data; in the writing of the manuscript, and/or in the decision to publish the results.

GC reports honoraria for speaker's engagement from Bristol Myers Squibb, Eli Lilly, Foundation Medicine, Gilead, Merck, Novartis, Pfizer, Roche, and Seagen; honoraria for consultancy from Ellipses Pharma, Roche, and Seagen; and honoraria for advisory board participation from AstraZeneca, Daiichi Sankyo, Eli Lilly, Foundation Medicine, Gilead, Novartis, Pfizer, Roche, and Seagen; ML: advisory role for Roche, Lilly, Novartis, AstraZeneca, Pfizer, Seagen, Gilead, MSD and Exact Sciences; speaker honoraria from Roche, Lilly, Novartis, Pfizer, Sandoz, Libbs, Knight, Daiichi Sankyo and Takeda; travel Grants from Gilead and Daiichi Sankyo; and Research Grant (to the Institution) from Gilead; all outside the submitted work.

MDM reports honoraria from AstraZeneca, Boehringer Ingelheim, Janssen, Merck Sharp & Dohme (MSD), Novartis, Pfizer, Roche, GlaxoSmithKline, Amgen, Merck, Takeda for consultancy or participation to advisory boards and direct research funding from Tesaro/GlaxoSmithKline, institutional funding for work in clinical trials/contracted research from Beigene, Exelixis, MSD, Pfizer and Roche.

S.C. is previous national AIOM president. Other authors declared to have no relevant financial or non-financial interests to disclose inherent to the paper.

## Funding

None.
