# Supplementary material for: Advancing treatment choices: CDK4/6 inhibitor switching in HR+/HER2- metastatic breast cancer
Source: Breast. 2025 Jan 10;79:103875. doi: 10.1016/j.breast.2025.103875 (PMC11786079; doi:10.1016/j.breast.2025.103875)
Supplement: Multimedia component 1 [file mmc1.docx]

1. In your experience, what is the percentage of patients who had to discontinue treatment with CDK4/6 inhibitors due to toxicity?
2. In your experience, what is the percentage of patients who had to discontinue treatment with palbociclib due to toxicity?
3. In your experience, what is the percentage of patients who had to discontinue treatment with ribociclib due to toxicity?
4. In your experience, what is the percentage of patients who had to discontinue treatment with abemaciclib due to toxicity?
5. What is the most common unacceptable toxicity that led to treatment discontinuation? (Please rank from 1 to 6, with 1 being the most frequent and 6 the least frequent):
   - Constipation
   - Asthenia
   - Hematologic toxicity
   - Hepatic toxicity
   - Abdominal pain
   - Other toxicities
6. **Do you think the possibility of switching CDK4/6 inhibitors in case of toxicity will influence your choice of the initial drug?**
   - Yes, significantly
   - Yes, moderately
   - Yes, but rarely
   - No, not at all
7. **If you answered "Yes" to the previous question, which CDK4/6 inhibitor would you choose differently compared to before? (You may select more than one answer)**
   - I would use more abemaciclib
   - I would use less abemaciclib
   - I would use more palbociclib
   - I would use less palbociclib
   - I would use more ribociclib
   - I would use less ribociclib
8. **The choice of the second-line treatment will most frequently be based on:**
   - Type of toxicity
   - Type of drug that was discontinued.
9. **Did this AIFA decision surprise you?**
   - Yes, very much
   - Yes, somewhat
   - No, not at all
10. **Do you agree with this decision?**

- Strongly agree
- Agree
- Neutral
- Disagree
- Strongly disagree.

1. **Do you believe there is sufficient evidence supporting the safety of switching CDK4/6 inhibitors?**

- A lot
- Enough
- Little
- No evidence

1. **Do you believe there is sufficient evidence supporting the efficacy of switching CDK4/6 inhibitors?**

- A lot
- Enough
- Little
- No evidence

1. **For a patient who was receiving the interrupted drug at a reduced dose, what dose would you start the new drug at?**

- Full dose
- Reduced dose, same as previous
- It depends on the case
- I don’t know, I would like more data

1. **If you have already switched CDK4/6 inhibitors, with what percentage of patients have you done so?**
2. **Which drug did you most frequently initiate as second-line therapy?**

- Abemaciclib
- Palbociclib
- Ribociclib

1. **How did the second CDK4/6 inhibitor perform in terms of toxicity?**

- Very well, no issues
- Quite well, there were problems but they were manageable
- Poorly, there were several problems and I had to discontinue
- Poorly, I had the same problems as before

1. **How much time passed between the toxicity-related discontinuation of the first CDK4/6 inhibitor and the initiation of the second one?**
2. **What was the average duration of treatment with the first CDK4/6 inhibitor and the second one after switching?**
3. **At what line of treatment was the patient at the time of the switch?**
4. **If the second CDK4/6 inhibitor was started at the full dose, did you need to reduce the dose with the second CDK4/6 inhibitor as well?**
